# Society Proceedings

**Published:** 1902-08

**Authors:** 


### SOCIETY PROCEEDINGS.

THE following important letters have been issued in connection with the Pennsylvania State Veterinary Medical Association, seventh annual meeting:

DEAR DOCTOR: The profession of Pennsylvania has just received the unique gift of the library of the late Dr. R. S. Huidekoper, through the generosity of one of our members, Dr. Thomas B. Rayner. It is a very important matter that this library should have a complete record of the profession's history and advancement as chronicled in our veterinary journals, and I write to ask you to send me copies of the various veterinary periodicals that have been published in our country during the last forty years, with a view of endeavoring to make up files of all these periodicals, have them suitably bound and placed with this valuable collection that is ours for reference and consultation, and which will remain a source of information and value to the future profession of our State.

Write me what you have and would be willing to give with a view of completing these records. Very truly yours,

W. L. RHOADS,
President.

DEAR DOCTOR: The semi-annual meeting of the Pennsylvania State Veterinary Medical Association will be held in the Board of Trade Rooms, No. 25 North Sixth Street, Reading, Pa., Tuesday, September 16, 1902, at 10 A.M.

There will be a number of papers read by some of the most progressive veterinarians in our State. The reports of cases also will be of especial interest. We trust you will lend your aid by being present and taking part in the various discussions at the regular meeting during the day.

Programmes of the meeting will later be sent to all our members and others who are interested enough to write for them.

The question of good roads is becoming most potent in Pennsylvania. It is of utmost importance to every taxpayer and every horse user or owner throughout the State. As President of the State Association, I feel that one of the greatest benefits we can bring to the horse raiser or user is to awaken an increase of interest in the matter of "good roads." This question, as exemplified by the profession, has not been made public enough, and in consequence of this I have decided to have an evening session (of our semi-annual meeting, to be held in the Board of Trade Rooms, No. 25 North Sixth Street, Reading, Pa., Tuesday, September 16, 1902), to be devoted to the consideration of "good roads."

I shall have to address us at this meeting some who are especially devoting their attention to its consideration, as well as members of our Association and the profession who are equally interested therein.

You cannot afford to miss this meeting, and we cannot afford to have you miss it; so I trust, to save us both, you will make a special effort to attend. Assuring you of a royal good time and a profitable experience, hoping to hear from you and trusting I may meet you and your friends who are interested in our work at Reading, September 16th, I am,

Fraternally yours,

W. L. RHOADS,
President.

### REPORT OF COMMITTEE ON SANITARY SCIENCE AND POLICE.[1]

By C. J. Marshall, V.M.D.,
PHILADELPHIA.

I have nothing of special interest to report as a member of this committee. There have been no outbreaks of contagious diseases in this section to require any special attention. There has been no decided effort to better or improve the system of city meat and milk-inspection. This subject has been conspicuous for the lack of effort that has been made.

The Keystone Veterinary Medical Society has appointed a committee to investigate the milk-supply of the hospitals and public institutions in the city. A circular letter has been sent to the superintendents of these institutions for the purpose of obtaining data to assist this committee in its work, which, when completed, we will hope will be of great benefit to the public institutions, at least in the matter of a better milk-supply.

The work being done by the Milk Commission of the Philadelphia Pediatric Society is progressing nicely. At present there are three dairies producing milk under its requirements, and this commission is certifying to its purity. One more good-sized dairy has pledged itself to furnish certified cream under the direction of the Milk Commission, and is to begin on the first of April. With but one exception, the producers have been able to fulfil the requirements of the commission the past year. The buildings on one farm were destroyed by fire, and for two months this producer was not able to satisfy the commission. We believe the work of this commission is along the right lines, and that it should receive all the encouragement and assistance possible from our profession. There is a chance for much improvement in this work. They have set the requirements high, yet there is no reason why they cannot be complied with if the efforts on the part of all interested parties are constantly applied. It seems necessary that the commission should furnish minute and explicit directions as to how all parts of the work shall be done. It is not sufficient to say that everything around the stable must be kept clean. The subject of cleanliness seems to be relative. One man's ideal is not that of another. It seems better to go into details on this subject and make certain definite rules how cleanliness must be obtained. As, for instance, the cows must be groomed once daily. The floors must be swept once each day and scrubbed once each week. The men shall wash their hands in soap and water and dry them on a clean towel and put on suitable clean milking suits before beginning to milk, etc. Rules from the commission are more easily enforced than those issued by the veterinarian or foreman.

We note with much pleasure the harmonious manner with which the State Live-stock Sanitary Board conducts its work and the increased confidence the stock raisers of the Commonwealth have in this board. It seems that it has become a necessity in the short time that it has been in existence. We believe that its rapid growth, as well as its birth and the respect in which it is held by the veterinarians as well as by every person

[1] Presented at the annual meeting of the Pennsylvania State Veterinary Medical Association, March 4 and 5, 1902.

who owns stock in this State, is due largely to the efforts of our State Veterinarian and the polite and skilful manner with which he conducts the work. It has not only benefited our profession in all sections of the State, but it can be held up as a model for other States to follow. We believe that there is no other State in the Union where this kind of work is better organized or its system more thoroughly executed. It brought about an amalgamation of the profession in our State. The success has been brought about largely through the skill, honesty, and perseverance with which each veterinarian has assisted in carrying out his part of the work. We should guard the good reputation of the State Live-stock Sanitary Board and the State Veterinarian with jealous watchfulness, and assist in every way possible to advance its usefulness.

Among the many features of the State Live-stock Sanitary Board we would note especially the bacteriological laboratory and our able bacteriologist, Dr. M. P. Ravenel, whose voice was heard at the International Congress on Tuberculosis, held in London last summer. He was able to refute the statement made by the mighty Koch, through the careful investigations made by Dr. Pearson and himself in the laboratory of our State Board. No government, national or State, is making more careful or strenuous efforts along the lines of investigating all phases of the subject of tuberculosis than is being carried on by these two men.

One of the most perplexing subjects in connection with dealing with bovine tuberculosis is, What shall be done with the tuberculous cattle? The board has found that the small appropriation made by the State is sufficient to pay for only a small percentage of the cattle that react to the tuberculin test. The farmers who have tuberculous herds are finding out that they cannot afford to get along without the tuberculin test. They cannot afford to lose the ones that react. The State cannot afford to pay for them, and the owner is not allowed to sell them.

We know that the reacting cows can be kept in such a way that they cannot spread the disease. We also know that the milk can be safely used from a large percentage of such cows under certain definite conditions.

It would seem just and reasonable that we use our influence to bring about some plan by which the maximum amount of usefulness can be harmlessly derived from these reacting cases.

This committee would recommend for the consideration of this Association the suggestion made by "Columella" in the January number of the JOURNAL OF COMPARATIVE MEDICINE AND VETERINARY ARCHIVES" for "the disposal of tuberculous animals." His suggestions are as follows: that certain persons be given a license to purchase and keep tuberculous animals, under conditions prescribed by the State Live-stock Sanitary Board. This license also permits such owners to raise the calves, and sell the milk and butter from such cows. We realize that this plan might be safely and profitably carried on; whether it is advisable to do so we leave for future consideration.

We would also compliment our Association and the profession at large for the speedy and effectual manner in which the Stiles bill was sidetracked since our last anual meeting, and that the requirements for admission to our ranks are still faithfully and watchfully guarded by the State Board of Veterinary Examiners. We recommend that this Association express its unqualified approval to the Governor, Hon. William A. Stone, for the good judgment exercised by him in the well-merited reappointment of our esteemed colleague, Dr. W. Horace Hoskins, as an active member of the State Board of Veterinary Medical Examiners.

### REPORT OF COMMITTEE ON ANIMAL HUSBANDRY.[1] (No. 1.)

By George B. Jobson, V.S.,
FRANKLIN, PA.

In the previous reports of this committee figures were given showing the importance of and the amount of wealth invested in the animal industry of Pennsylvania. While these figures giving the numbers and estimated value of the horses, cattle, sheep, and swine in this State show the immense capital invested in live-stock, it is not merely from a commercial or financial standpoint on which we must base our estimate of the relative importance of this industry to the citizens of our Commonwealth. When we take into account the fact that the wholesomeness of the products derived from our domesticated animals, in the form of meat, butter, and cheese, is largely dependent on their health, their sanitary surroundings, and methods of handling these products, we begin to appreciate in some measure the importance of the animal husbandry in relation to the health of the consumer.

We endeavored to demonstrate the position which the veterinary profession holds to the animal husbandry of this Commonwealth—that the mission of the veterinarian, as formerly understood by his client, lies not so much in curing the diseases of animals as in their prevention and control, by advising the owner regarding the enforcement of efficient sanitary and hygienic measures which shall prevent the approach of or control the spread of disease.

The reports issued by the agricultural department of the several States and the Bureau of Animal Industry are all prominent factors in educating the farmer regarding the necessity of adopting such measures as shall provide for the comfort and sanitary condition of his live-stock; and to keep in line with these improved educational advantages enjoyed by our agricultural friends the education of the successful veterinarian in rural districts will necessarily require to embrace a course of study which shall enable him to give intelligent advice in regard to the sanitary housing, feeding, and general care of farm stock. While probably not so essential, a knowledge of the good qualities and points of the various breeds is a very useful qualification.

The State Live-stock Sanitary Board is still fighting the good fight for the control and stamping out of bovine tuberculosis. The badly infected herds having been first inspected, those now being tested show a much smaller percentage of diseased animals. While the public generally appreciate the good work which is being done by the board for the control of tuberculosis within our own borders, and preventing the entry of untested dairy and breeding stock into this State, yet there are a few noisy and carping critics who make wholly irresponsible and unfounded statements regarding cattle inspection and the tuberculin test, which only go to show their own ignorance of the utility of this test for the detection of tuberculosis.

[1] Presented at the annual meeting of the Pennsylvania State Veterinary Medical Association, March 4 and 5, 1902.

It is to be deplored that a few of our live-stock journals are still prejudiced against the tuberculin test as a means for the diagnosis of tuberculosis. A recent communication in one of these journals by a veterinarian resident in a neighboring State was not only a breach of professional courtesy, but a gross insult to the veterinarians of Pennsylvania.

There does not appear to have been any severe epizootic disease among live-stock since our last report. A form of hemorrhagic septicæmia occurred in a herd of dairy cattle near Corry, during the latter part of September, and at the same time in a herd in Warren County. It was characterized by severe hemorrhagic diarrhœa, elevated temperature, and rapid respiration, and in the majority of the animals affected proved fatal in a few hours. At both places the cattle were pastured on poor, unreclaimed land, partly in the woods, and the water-supply was bad, being the surface drainage from the land. Removal to other quarters and feeding corn fodder stopped further extension of the outbreak. On suspicion of anthrax, specimens from animals affected in both herds were forwarded to the bacteriological laboratory of the State Live-stock Sanitary Board, but the results were reported negative. One or two of the last animals affected, which lingered for some weeks after being attacked by the disease, were slaughtered for post-mortem examination. The lesions found in this case were only those of simple gastro-enteritis. The abomasum, pyloric orifice, and intestine adjoining the organ were intensely inflamed on their mucous surface. The ileocæcal valve and colon in spots were also affected in the same manner. The débris of dried leaves in considerable quantity, along with corn fodder which had been lately fed, were found in the rumen.

The demand for dairy inspection by municipalities is increasing, although much remains to be done by way of educating the public regarding the advantage of inspection in providing a pure and wholesome milk-supply for consumers. When a system of milk-inspection is first introduced in a district it is well to make the requirements not to exacting, but gradually lead up to that point which will give a good and efficient system of inspection. The difficulty in most cases is not that the milk is of poor quality, but in impressing on farmers and dairymen the necessity for providing enough air space, plenty of light, keeping the stable in good sanitary condition, and having proper conveniences and methods for cooling and handling the milk.

It is with pleasure we note the demand for and good prices realized for all classes of farm stock, horses, fat cattle, dairy stock and its products, and the generally improved financial condition of the farmer. Usually with this financial improvement the agriculturist desires to improve the quality of his live-stock. This increased value creates a demand for the services of the veterinarian when they are attacked by disease, which proves how closely the prosperity of the veterinarian is dependent on that of the farmer.

### REPORT OF COMMITTEE ON ANIMAL HUSBANDRY.[1] (No. 2.)

This is a subject of great importance to us. If animal husbandry fails our practice is gone.

[1] Presented at the annual meeting of the Pennsylvania State Veterinary Medical Association, March 4 and 5, 1902.

No legitimate industry can suffer without rebounding to the injury of society in general, and most certainly none can take rank ahead of the industry that affords the human family its meat and milk-supply as well as the chief part of the power used in tilling the ground, storing, preparing, and moving the crops, delivering the articles of commerce to the doors of our homes, and moving the engines of destruction and our rough riders in time of war.

From the careful investigation of the *American Agriculturist* we find a loss of 4 per cent. in farm stock values during the twelve months from January, 1901, to January, 1902, the total shrinkage being over $125,000,000, notwithstanding a moderate increase of numbers in all classes of animals except hogs.

The average price of horses has declined 2 per cent., mules 1 per cent., cows 5 per cent., cattle 11 per cent., sheep 10 per cent., while hog values have advanced 17 per cent. If this condition prevails only in the drought-stricken districts we might consider the cause local, but in every State the decline is noticeable and quite uniform. Of course, the high price of feeds and forced sales of stock have had their influence; but, aside from these, there are plain indications that the upward trend of live-stock values is over and on the decline. We hope and trust they will not again reach the ruinous prices of 1896.

The average price of hogs increased $1.11 per head the past year, making a bright prospect for the hog raiser who is well stocked and well posted in economical production. Prices will doubtless be well maintained for at least one year, owing to the unusual slaughter of store hogs and brood sows causing a light pig crop this spring. In fact, anomalous conditions are present among the other meat producers (cattle and sheep).

Cattle known as stockers can be bought lower than at any time in recent years, while prime beef cattle and veal calves are at top notch. Store sheep and lambs have ruled low, while the well-fattened carcass markets well, giving the man who has capital and understands the science of feeding a chance to make money. It is true that the farmer has shown prudence in hesitating about feeding on corn alone this winter; but cottonseed and linseed meals and the gluten foods are proportionately lower and can be profitably used, with corn, in making beef, pork, and mutton, while the value of the manure heap is greatly enhanced.

The Eastern farmer who raised one hundred bushels of corn to the acre and laid in a good supply of nitrogenous foods during the summer and early autumn has been in position to turn the markets to good advantage.

The feeding problem is of first importance in animal husbandry, and the veterinarian should be the farmer's ready adviser under the varying conditions.

### REPORT OF SEMI-ANNUAL MEETING OF THE VETERINARY MEDICAL ASSOCIATION OF NEW JERSEY.

THE semi-annual meeting of the Veterinary Medical Association of New Jersey was held at Statter's Assembly Hall, No. 842 Broad Street, Newark, on Thursday, July 10th.

The following members responded to the roll-call:

Drs. A. W. Axford, A. Brown, T. Earle Budd, D. J. Dixon, J. M.

Everitt, J. B. Finch, W. J. Fredericks, J. O. George, James T. Glennon, G. P. Harker, W. F. Harrison, R. C. Hasbrouck, E. A. Hogan, J. B. Hopper, B. F. King, E. L. Loblein, Seth Lockwood, William Herbert Lowe, A. P. Lubach, Charles T. Magill, E. Mathews, James T. McDonough, James M. Mecray, John M. Mitchell, John P. Mathews, E. F. Meiners, E. R. Ogden, George W. Pope, Werner Runge, T. E. Smith, A. T. Sellers, M. M. Stage, S. S. Treadwell, L. E. Tuttle, and H. Vander Roest.

Hon. S. B. Ketcham, of the State Tuberculosis Commission, and Dr. Samuel Glasson, veterinarian in the United States army, and on a two months' leave of absence after a term of service in the Philippines, were in attendance as guests of the Association.

The following approved applications for membership were in the hands of the Secretary:

Dr. Phineas Bridge, of Montclair; Dr. G. Walter Dilkes, of Mullica Hill; Dr. John L. McCoy, of Sussex; Dr. John C. Peterson, Jersey City; Dr. T. B. Rogers, Woodbury; Dr. George B. Vliet, Hackettstown; and Dr. Thomas H. Ripley, Newark.

Upon ballot the above were unanimously elected to membership.

Drs. Bridge, Dilkes, Rogers, and Vliet were present, and were introduced by President Lowe.

Great interest centred in the report of the Committee on Legislation, and as Dr. Budd, chairman, recounted the work of the committee and the efforts put forth on behalf of the bill which has now become a law and is for the regulation of the practice of veterinary medicine, surgery, and dentistry in the State of New Jersey, no one present could fail to be impressed with the fact that in organization there is power if in the organization there be a common aim and men who are willing to sacrifice time, money, and energy without expectation of personal aggrandizement.

The impression prevails that the story was not half told by Dr. Budd. Men who achieve are usually endowed with a corresponding modesty, and it must be left to others than the members of this committee to fully narrate the difficulties met, the tact displayed, and the untiring energy exhibited by those to whom the Association entrusted this important measure.

He would be a dull man who, in the light of the passage of this act, could not see the veterinary profession of New Jersey reaching a higher plane. He would be a poor Association member who, after listening to the report of the Legislation Committee, did not "enthuse" or feel proud to be affiliated with an organization which stood for advancement, and thus may be explained a feeling of optimism and dignity which pervaded the meeting held at Newark on the 10th.

After the report of the Legislation Committee had been received with thanks, Dr. Lowe was called from the room, and with Dr. Budd in the chair a purse was raised and a committee dispatched to purchase a sterling silver dinner set for presentation to Dr. Lowe in recognition of the effort put forth by him in behalf of the recently enacted law. The presentation was made at the close of the banquet, and will be referred to later.

Under "new business" Vice-President Budd again assumed the chair, in order to enable President Lowe to present several matters, which are here given as presented to the Association.

*Recognition of the Passaic County Veterinary Medical Association.*
*(On motion of Dr. Lowe.)*

MR. PRESIDENT: I take great pleasure in reporting that ten regular practitioners of veterinary medicine resident in Passaic County met at my office in the city of Paterson, on Monday evening, July 7, 1902, and organized a Passaic County Veterinary Medical Association.

It is the intention and purpose of this organization to be in affiliation with the Veterinary Medical Association of New Jersey, and it was resolved that the Passaic County Veterinary Medical Association should be to the veterinary profession of the county what this Association is to the profession of the State.

The members of the local organization pledged themselves to do all in their power as individuals and as members of the Society to advance and promote the common interests of the profession in the county of Passaic.

The veterinarians of the various counties, wherever there are a sufficient number of practitioners to warrant it, should organize county societies in their respective counties. Nothing, in my opinion, would strengthen the State Association more than live county societies.

The organization of the Passaic County Veterinary Medical Association, I predict, is only the beginning of a movement to establish similar local organizations throughout the respective counties of the State. Mr. President, I have the honor to announce the Passaic Veterinary Medical Association, duly organized in said county, as the first born. I now move you, in behalf of the local organization, that the Veterinary Medical Association of New Jersey now in convention assembled officially recognize, declare, and accept the said Passaic County Veterinary Medical Association as the county organization of the county of Passaic, in the State of New Jersey, with all the rights and privileges belonging to or appertaining to a county association, so long as nothing in its Constitution, By-laws, or Code of Ethics shall be inconsistent or conflict with the Constitution, By-laws, or Code of Ethics of the Veterinary Medical Association of New Jersey. The motion was unanimously carried.

Dr. Lowe then spoke of the tireless and able efforts of Senator Wood McKee, which rendered possible the recent veterinary legislation in the State; also reference was made to the evident approval, on the part of Governor Murphy, of the efforts of the Association to secure proper and necessary veterinary legislation. It was moved and carried that the by-laws be suspended and the above gentlemen elected honorary members of the Veterinary Medical Association of New Jersey.

*Minneapolis Party Committee. (Motion by Dr. Lowe.)*

"It is fresh in our minds how, only last September, the representative veterinarians of America journeyed from far and near to Atlantic City, upon invitation of the Veterinary Medical Association of New Jersey, to attend the international veterinary convention held at this mecca by the sea.

"A cordial and hearty invitation is now extended by the veterinarians of Minnesota to the veterinarians of New Jersey to visit them on the occasion of the forthcoming annual meeting of the American Veterinary Medical Association at Minneapolis, September 2d, 3d, and 4th. The wives and families of members are included in this invitation. The social features of the annual meetings of the American Veterinary Medical Association are becoming greater and greater every year.

"Specialists in every phase of veterinary science, as well as the general practitioner, will find much at this meeting of value and interest to them. In fact, no progressive up-to-date practitioner can afford not to attend this great veterinary meeting in September. Special railroad rates will be allowed. Let us, with the neighboring States, form an Eastern party and charter a car. I move, Mr. President, that a Minneapolis Party Committee be appointed, and that this committee be authorized to form a Minneapolis party which shall include veterinarians of neighboring States who may desire to join the party, and if a sufficient number pledge themselves to join the party to warrant it, to make arrangements and charter a special car."

The motion was carried, and the following were appointed a committee: Drs. T. E. Smith, of Jersey City, James M. Mecray, of Maple Shade, and G. F. Harker, of Trenton.

This committee was able to report before the meeting adjourned that a rate of $53.35 for the round trip, New York to Minneapolis and return, had been quoted to them by one of the transportation lines, and that the above amount would include accommodations in the sleeping-car, providing eighteen would attend.

The enactment of the new State law made necessary a change in the Constitution, and accordingly an amendment was proposed and came up for first reading. This amendment provides that candidates for membership entering the profession on or after the first Monday in May, 1902, must be licensed by the State Board of Veterinary Medical Examiners and be registered in conformity with the provisions of chapter xviii., Laws of 1902.

Hon. S. B. Ketcham addressed the members on the growing need of well-educated and qualified veterinarians, and urged that the efforts of the Association to secure an educated and competent line of practitioners be continued in the future as it had been in the past.

Recognition of the veterinary profession in the military service was discussed, and the President urged that action be taken toward having it fittingly recognized in rank and title. The Secretary was instructed to communicate with the military officials at Trenton, calling attention to this condition.

At 1.30 P.M. adjournment was made for dinner, and about fifty members gathered about the board in Stetter's dining hall. At the conclusion of the dinner Dr. T. B. Rogers, on behalf of the fellow-members, presented Dr. Lowe with a gift of dinner-table silver. In making the presentation, Dr. Rogers said in part: "Dr. Lowe, you found us a scattered profession; you have bound us together. You found us without the pale of the law; you have placed a protecting arm around us. In behalf of the profession you have united, I have great pleasure in offering you this little token of our regard."

At the afternoon session Dr. James T. McDonough's paper, entitled "The Horse's Foot," was discussed at length. Dr. McDonough proved his statements by a practical demonstration, and answered all objections in a clean-cut manner which strengthened the general impression that he is an authority upon lameness and shoeing.

Owing to the lateness of the hour, Dr. James M. Mecray's paper upon "Some of the Necessary Qualifications for Producing Wholesome and Clean Milk" was not read, it being voted that the paper be presented by Dr. Mecray at the next meeting.

A ballot was taken to decide upon the place of the next meeting. Some favored Lakewood and others Jersey City, but the majority of the ballots were cast for Trenton, and later the vote was made unanimous that the next meeting be held at Trenton on the second Thursday in January, 1903.

At 4 P.M. the meeting was adjourned to the Newark City Hospital where Dr. Werner Runge, veterinarian of the Newark Board of Health, gave a demonstration of the methods of producing antitoxin serums, and Dr. T. B. Rogers performed three neurectomy operations. A vote of thanks was extended to Dr. Runge, Dr. Rogers, and the hospital management for their kindness in making the clinic such a successful feature of the meeting, and the members departed for their homes well satisfied that the day had been a profitable one.

George W. Pope,
Secretary,

### PASSAIC COUNTY VETERINARY MEDICAL ASSOCIATION.

Drs. W. J. Reagan, M. A. Pierce, Alexander Machan, David Machan, T. J. Cooper, Harry K. Berry, and William Herbert Lowe, all of Paterson; Dr. George W. Pope, of Athenia; Drs. A. P. Lubach and J. Payne Lowe, of Passaic, met at Dr. William Herbert Lowe's office, corner of Paterson and Van Houten streets, Paterson, N. J., at 8 P.M., on Monday evening, July 7, 1902, in response to a call for a meeting of the veterinarians of Passaic County for the purpose of organizing a Passaic County Veterinary Medical Association.

Dr. William Herbert Lowe called the meeting to order at 8.30 P.M., stated the object of the meeting, and briefly outlined the advantages and benefits of a county society. It was moved and carried that a county organization be formed, and that a President, First Vice-President, Second Vice-President, Secretary, and Treasurer be elected. The election resulted as follows: President, Dr. William Herbert Lowe; First Vice-President, Dr. David Machan; Second Vice-President, Dr. T. J. Cooper; Secretary, Dr. Alexander Machan; Treasurer, Dr. M. A. Pierce.

It was regularly moved and carried that an Executive Committee of five be appointed by the Chair. The Chair appointed on such committee Dr. George W. Pope, Chairman; Dr. Harry K. Berry, Dr. Anthony P. Lubach, Dr. William J. Reagan, Dr. J. Payne Lowe.

Dr. David Machan moved that the next meeting be held July 14th, and that the County Association meet monthly thereafter; that the regular monthly meetings be held on the second Monday evening of each month. Carried.

On motion of Dr. Alexander Machan, the meeting adjourned to meet Monday evening, July 14th, at the same place and hour.

An adjourned meeting of the Passaic County Veterinary Medical Association was held at Dr. Lowe's office, Paterson, N. J., on Monday evening, July 14, 1902.

President Lowe called the meeting to order at 8.30 o'clock.

Dr. David Machan was requested to act as Secretary in the absence of his brother. The following practitioners of Passaic County answered to their names:

Dr. Harry K. Berry, Paterson; T. J. Cooper, Paterson; John H. De Graw, Paterson; William H. H. Doty, Paterson; William C. Ferguson, Paterson; M. A. Pierce, Paterson; George W. Pope, Athenia; William Herbert Lowe, Paterson; J. Payne Lowe, Passaic; A. P. Laubach, Passaic; David Machan, Paterson; William J. Reagan, Paterson.

Dr. Fredericks, of Delawanna, telephoned that he had been detained and could not reach the meeting in time, and requested the President to announce to the meeting that he would stand by whatever the majority did at the meeting.

Dr. Berry stated that he had received a letter from Dr. Brooks (who is away on a vacation), and that he expressed himself as heartily in favor of the movement.

The President reported that the Veterinary Medical Association of New Jersey, at its semi-annual meeting in Newark, on the 10th instant, had passed resolutions officially recognizing the local organization as the Passaic County Veterinary Medical Association, duly constituted as such in full affiliation with the State Association. The State Association congratulated the veterinarians of Passaic County on starting the movement and on being the first to organize a County Association in the State, and expressed the hope that the practitioners of other counties, whenever and as the number of veterinarians would warrant it, would form county associations in their respective counties.

Dr. Reagan moved that the Chair appoint a Committee on Constitution, By-laws, and Code of Ethics, which was carried.

The Chair appointed on such committee Dr. Ferguson, Chairman; Dr. Doty, Dr. Reagan.

Dr. Doty moved that the Chair appoint a special committee to prepare and present a table of fees and rates of charges for professional services, on similar lines with the table of fees of the Medical Society of New Jersey, for the government of the members of this Association. Carried.

The Chair appointed on such committee Dr. J. Payne Lowe, Chairman; Dr. Fredericks, Dr. De Graw.

Dr. Cooper moved that the matter of making a black list of "dead beats" be taken up at the next meeting. Carried.

Violations of the provisions of the new veterinary law (chapter xviii., Laws of 1902) were reported; and as the said enactment prohibits all persons not registered before the first Monday in May, 1902, from entering upon or continuing the practice of veterinary medicine, surgery, or dentistry, in any of their branches, in the State of New Jersey without being licensed by the State Board of Veterinary Medical Examiners, and registered at the county clerk's office, in conformity with the provisions of the act it was decided to procure evidence of violations in this county for the purpose of prosecuting offenders. The Association decided to furnish evidence and otherwise aid the State Board of Veterinary Medical Examiners in convicting persons guilty of violating any of the provisions of chapter xviii. Laws of 1902.

It was moved and carried that Dr. J. Payne Lowe be requested to prepare and present a paper on "Veterinary Ethics" at the September meeting of the Association (September 8, 1902).

On motion, the meeting adjourned to meet at the next regular meeting night (Monday, August 11), at the same time and place.

This is to certify that we, the subscribers, practitioners of veterinary medicine, surgery, and dentistry, of the County of Passaic, State of New Jersey, upon the invitation of Dr. William Herbert Lowe, met at his office, corner of Paterson and Van Houten streets, Paterson, N. J., on Monday evening, July 7, 1902, and organized a Passaic County Veterinary Medical Association, and that it is the intention and purpose of this organization to be in affiliation with the Veterinary Medical Association of New Jersey, incorporated April 15, 1885, under an Act of the Legislature for the promotion of veterinary science and art. It is hereby resolved that this Society shall be to the veterinary profession of Passaic County what the Veterinary Medical Association of New Jersey is to the veterinary profession of the State.

We do hereby further pledge ourselves to do all in our power as individuals and as members of this organization to advance and promote the common interests of the profession in this county.

William Herbert Lowe,
David Machan,
T. J. Cooper,
Alexander Machan,
M. A. Pierce,
George W. Pope,
Harry K. Berry,
Anthony P. Lubach,
William J. Reagan,
J. Payne Lowe.

Signed July 14, 1902.
W. H. H. Doty,
William C. Ferguson,
John H. De Graw.

William Herbert Lowe,
President.
David Machan,
Secretary.

The regular monthly meeting of the Passaic County Veterinary Medical Association was called to order by President William Herbert Lowe at 8.30 p.m., August 11, 1902, when Dr. David Machan was chosen Secretary *pro tem.*

The following members answered to their names on roll-call: Drs. William J. Reagan, David Machan, Harry J. Berry, William H. H. Doty, William C. Ferguson, T. J. Cooper, M. A. Pierce, John H. De Graw, William H. Lowe, Jr., and William Herbert Lowe, of Paterson; J. Payne Lowe and R O. Hasbrouck, of Passaic; George W. Pope, of Athenia; William J. Fredericks, of Delawanna.

A telegram was read from Dr. William C. Berry, of Bloomingdale, expressing his inability to be present.

The minutes of the last meeting were read and approved.

The President called for the report of the Committee on By-laws and Code of Ethics. Drs. Ferguson and Reagan, of the committee, reported that the By-laws and Code were in course of preparation, but not yet completed, when, upon a motion made by Dr. Hasbrouck, the adoption of the same was laid over until the next meeting.

Dr. J. Payne Lowe, chairman of the Committee on Schedule of Fees, presented a carefully prepared report, signed by all members of the committee—Drs. J. Payne Lowe, William J. Fredericks, and John H. De Graw —which, upon motion of Dr. Reagan, was fully discussed, and the schedule amended, and adopted as amended, by a unanimous vote, and the schedule was ordered printed and a copy furnished each member, all agreeing to govern themselves by the said schedule. The report and schedule as finally adopted follows:

Mr. President and Gentlemen: The circumstances under which veterinary services are rendered vary greatly, and while we have made up and will submit a schedule of rates to be charged, we believe practitioners should use discretionary power in charging. Some men can command larger fees than others; men of reputation and experience more than young veterinarians. We should adopt no radical tariff of fees that will make us or our services unpopular with the public, or that will have a tendency to cause them to do without our services and to resort to empirical measures.

Our services are sought for, as a rule, for commercial reasons. We not infrequently treat very valuable animals. As a rule, however, most of us are called upon to save the life of a $200 horse; to restore to usefulness a $75 lame horse; to assist a $50 or $60 cow at the time of parturition. True, quite a few animals are treated where this does not obtain. The family horse, on account of his docile qualities, is often treated to an extent not in keeping with his market value; or occasionally it is an old animal that has been pensioned off until of necessity he is humanely destroyed; or, again, in canine practice it is often a matter of sentiment. So you see in practice it is not what we should receive for some fancy operation skilfully performed, but we must keep within practical bounds; in other words, the value of the animal treated must be considered. In cases covering a long period of time or terminating unfavorably it is not only fair but it is policy sometimes to make concessions. The volume of work we do for a client should modify the fees; or, again, poor people should be charged leniently or in extreme cases not charged, while those able to pay should be charged the full fee.

Your committee does not believe that there is any member of the Passaic County Veterinary Medical Association who would lower his dignity or undermine his brother practitioner by under-charging for his professional services, and the schedule of fees we have made up is merely a guide to fellow-members as to what we believe to be proper charges, and we respectfully submit it for your consideration.

*Schedule of Fees—General Practice.*

Single visit in the city, $2.

Ordinary visits within the city, $1 to $2.

Ordinary visits outside the limits of the city, $2.50 and mileage.

Mileage at the rate per mile (according to the number of miles), 25 cents to 50 cents.

Advice given each additional animal at same visit, 50 cents to $1 extra.

Visits after 9 P.M. or before 7 A.M., or in haste, or in extraordinary circumstances, to be charged double.

Remaining in attendance all night, $10 and upward.

Detention in addition to visit, per hour, $1 to $2.

*Surgical Practice.*

Ordinarily, visits the same as in general practice, or double if surgical dressings, etc., are applied.

Minor operations, $1 and upward.

Dressing abrasions, sores, etc., $1 and upward.

Suturing and dressing wounds (trifling in character), $2 and upward.

Blisters applied, $2 and upward.

Insertion setons, $2 to $3.

Phlebotomy (jugular), $2 to $3.

Major operations, $5 and $10 and upward.

Suturing and dressing wounds (serious in character), $5 and upward.

Neurectomies, plantar (high or low), $10 per foot and upward; median, $15 per foot and upward; tibial, peroneal, $15 to $25.

Operations for the removal of champignon, $10 and upward.

Amputation of the penis, $10 and upward.

Tracheotomy, $5 and upward.

Use of the actual cautery, $5 and upward.

Fractures (setting of fractured limbs, etc.), $2 and upward.

Castration of the male, single horse (normal), $10; two horses (normal), $15; three horses (normal), $20; four horses (normal), $25; cryptorchid, $25; bull, $3 to $5; dogs and goats, $2 and upward; cat, $1 and upward; pigs (single), $1 and upward; each additional pig, 50 cents.

Ovariotomy, mare, $25; cow, $10; bitch or cat, $5; sow, $3; each additional animal, $1.

Obstetrical practice, delivering fœtus, dystokia cases, by natural passages: mare, $10; cow, $5; smaller animals, $2 and upward.

Cæsarean operation: mare or cow, $10 and upward; smaller animals, $5.

Embryotomy: mare or cow, $10 and upward.

Removal of placenta: mare or cow, $3 and upward.

Replacing everted uterus: mare, $5 and upward; cow, $3 and upward.

Uterine douches, $2.

*Dental Operations.*

Filing molar teeth (under ordinary circumstances): one horse, $2; two horses, $4; in large stables after the first two horses, $1 per head.

Cutting elongated molars, $3 to $5.

Cutting incisors, $3.

Extracting wolf teeth, 50 cents each.

Extraction of temporary incisors, $2.

Extraction of molars, $5 and upward.

Trephining sinuses, $10 and upward.

*Miscellaneous.*

Consultation in all cases, $5 and upward.

Examination of horses for soundness, $5 each.

Post-mortem examinations, $5 each.

Chemical, microscopical, and bacteriological examination of urine, milk, etc., $5 and upward.

Chemical analysis in cases of poisoning, $5 and upward.

Opinion as an expert, $5 and upward.

Professional certificates, $2 and upward.

Assistant surgeon is entitled to a fee equal to one-half of that charged by the surgeon.

*Office Practice.*

Advice, $1.

Advice by letter or by telephone, $1 and upward.

*Medicines.*

Liniments, lotions, solutions, mixtures, etc., 50 cents to $1.

Ointments, 50 cents to $1.

Powders, per package, 50 cents to $1.

Signed by the committee:

J. PAYNE LOWE, *Chairman*,
WILLIAM J. FREDERICKS,
JOHN H. DE GRAW.

On motion by Dr. Hasbrouck, seconded by Dr. Pierce, the date of the regular monthly meetings was changed from the second Monday evening of the month to the first Tuesday evening of each month, except in the month of September, when the regular meeting shall be held on the third Tuesday evening.

President Lowe stated that the *American Veterinary Review*, in the August number, had given three pages of its space to a report of the organization and the proceedings of the Passaic County Veterinary Medical Association, and an editoral that would make every veterinarian in Passaic County feel proud that he belonged to the local organization.

The President called the attention of the members to the fact that the State Association had at its last meeting appointed a Minneapolis Party Committee, of which Dr. T. E. Smith, 309 Barrow Street, Jersey City, is chairman, and that the County Association should be represented at the meeting of the American Veterinary Medical Association in Minneapolis; and that any member or members that could go were invited to join the party going from this State on Saturday, August 30th. Dr. Cooper asked the President to represent the local organization, and, upon his motion, Dr. Lowe was elected the official delegate to the Minneapolis meeting.

It was also the sense of the meeting that the County Association should be represented at the forthcoming annual meeting of the New York State Veterinary Medical Society, to be held in Brooklyn, September 9th and 10th. Dr. Pierce moved that the Chair appoint delegates to the said meeting. The Chair stated that he would appoint as such delegates such members as expected to be able to attend the meeting. After the conference the Chair appointed delegates as follows: Drs. J. Payne Lowe, William H. Lowe, Jr., and William C. Ferguson.

The President requested practitioners who have students with them to advise them that the State Board of Veterinary Medical Examiners, under the law, would only recognize diplomas from colleges with a curriculum of at least three years.

On motion, meeting adjourned.

WILLIAM HERBERT LOWE,
President.

DAVID MACHAN,
Secretary *pro tem.*

### THE ILLINOIS VETERINARY MEDICAL AND SURGICAL ASSOCIATION.

THIS Association met in semi-annual session at the Brunswick Hotel, Decatur, August 14 and 15, 1902. The meeting was called to order by the President, Dr. V. G. Hunt, of Arcola, Ill. The roll-call was responded to by a fair number of the members.

President Hunt's address was delivered in a masterly manner, characteristic only of himself, and was as follows :

"GENTLEMEN : As we meet to-day we are forcibly reminded of the rapid advancement in every department of the arts and sciences. When we look back a quarter of a century ago and see where the veterinarian stood, the meagre supply of veterinary literature at his command, the impotency of the healing art, it is not to be wondered at that he should make some fearful mistakes. The only wonder is that he did as well as he did. In surgery no antiseptic precautions were thought of. Still, many of our patients recovered. One fact yet remains to be told. Very few of those bold and difficult operations were not attempted that to-day are everyday occurrences. Surgery has outstripped all the other sciences, has attained a degree of perfection never dreamed of a half century ago. Doubtless if we could brush aside the veil that covers the future we would still see greater surprises in store than ever before. The live-stock interest is too great to leave a stone unturned to combat every infectious and contagious disease that has already secured a foothold in our own State.

"We are well aware that an alarmist is not looked upon with any degree of composure. Still, this does not relieve the veterinarian of his responsibilities to his patrons. The time was when a lone horseman at breakneck speed warned the people of Johnstown of impending danger, losing his own life to save that ill-fated city ; they heeded not the warning, and the city was destroyed.

"Tuberculosis has come to stay. It has stealthily invaded every part of our State, slowly but surely doing its work ; and man can and does contract this disease from the bovines. Is it not worth while to give this disease our earnest consideration? Laws should be passed to restrict its ravages ; ample appropriations made for the experimental stations, with competent men at the helm. How important it is for politics to be eliminated from these appointments—not a 'good, clever fellow,' which usually means a d—d fool—but conscientious men of education, coupled with experience ; then we may expect results. How often has the worthy veterinarian skulked off in shame when a State Veterinarian has been called upon, and when confronted with a contagious or infectious disease was as dumb as an oyster. Let us hope we have seen the last of this class of gentry. If there is anything that requires keen perception, with a little common honesty, it is the position of State Veterinarian. It must be admitted the life of a veterinarian is an arduous one, above all the other vocations in life, for the public look with no degree of composure upon his shortcomings, while the human practitioner can attribute a death to 'heart failure,' an almost meaningless term, which is received in good faith.

"Our societies should sound the timely warning from the housetops without fear, favor, or affection. He should devote his whole life to the upbuilding of his self-chosen profession. As the practice of medicine is an incomplete science, and will likely always remain so, it requires a practitioner to be a diligent student while attending his everyday practice. Hence the necessity for our society. Life is too short to gain that perfection required unaided by the society. The man who gets what he has learned along the journey of life and lets it be buried with him is not a benefactor of his race. Let us encourage every worthy investigator. Be slow in our criticisms. 'With charity for all, with malice toward none,' is a commendable virtue. I thank you for your courtesy and valuable aid."

The minutes of the previous meeting were then read and approved by the Association.

President Hunt next appointed S. H. Swain, C. A. Hurlbutt, and John Osborne as a committee to draft resolutions of condolence on the death of Dr. L. C. Pray, of Minonk, Ill., a charter member of the Association, and directed that a copy thereof be sent to the widow of the deceased, and also one to be spread upon the minutes of this Association. The resolutions on the death of Dr. L. C. Pray follow:

WHEREAS, By the death of Dr. L. C. Pray the Association has been deprived of one of its brightest and most faithful workers; therefore, be it

*Resolved*, That in the late Dr. L. C. Pray we recognize a man of learning and character and an able practitioner of his profession; that we shall remember him as a man of noble impulses, of genuine and kindly sympathy with his fellow-men, of a warm heart and honest mind;

*Resolved*, That we tender our sympathy and condolence to the bereaved wife and family of the deceased in the hour of their affliction;

*Resolved further*, That a copy of these resolutions be sent to the family of the deceased, and a copy be spread upon the minutes of this meeting.

S. H. SWAIN,
C. H. HURLBUTT,
JOHN OSBORNE,
Committee.

The following papers were read: "Lymphangitis," by Dr. N. P. Whitmore, of Gardner, was of much interest. "Dermatorrhagia" was a subject of great interest, and Dr. C. A. Hurlbutt, of Stonington, read a report of a case in his practice which brought forth a very instructive discussion upon the subject. Dr. W. J. Martin, of Kankakee, next read a most able and instructive paper on "Bone Spavin: Its Etiology and Treatment," which paper was read before the American Veterinary Medical Association and published in the proceedings of that meeting. A very excellent paper was read by Dr. J. W. Marsh, of Illiopolis, on the subject of "Erysipelas," which brought out some very interesting and instructive points with reference to this disease. He was responded to by Drs. J. M. Reed and I. M. Luzader. The subject of "Nephritis" was well calculated to bring forth some very interesting discussions, and an excellent paper on the subject was read by Dr. J. M. Reed, of Mattoon, responded to by Dr. D. K. Gooddale. (Will appear later in the JOURNAL.)

The second day's session was called to order by President Hunt. Much discussion was indulged in on the subject of eligibility of members, and resulted in the carrying of a motion, proposed by Dr. S. H. Swain, to empower the Committee on Programme to draft and have printed a circular letter to be sent out to the profession at large in the State.

Dr. S. H. Swain next read a paper on the subject of "Ectropion and Entropion," which was responded to by Dr. R. W. Brathwaite. The subject of "Eczema" was next on the programme, by Dr. V. G. Hunt, of Arcola, who read an instructive paper, which was responded to by Drs. R. W. Brathwaite and C. A. Hurlbutt. "Tetanus," by Dr. R. W. Brathwaite, of Champaign, was a very complete and instructive paper, which created considerable interesting discussion. Dr. W. A. Swain then gave an account of "Hysteria," which was responded to by Dr. W. J. Martin.

Among the visitors present was Dr. Tiffany, of Springfield, State Veterinarian.

On motion, the date and location of the next meeting was fixed for Wednesday and Thursday, January 14 and 15, 1903, at the St. Nicholas Hotel, Decatur, Ill.

W. A. SWAIN,
Secretary.

### GENESEE VALLEY VETERINARY MEDICAL ASSOCIATION.

THE semi-annual meeting of the Genesee Valley Veterinary Medical Association was held at Rochester, July 17, 1902.

President O. B. French presided. There were present Dr. J. H. Taylor, Henrietta; P. I. Johnston, Wilmington; J. E. Smith, Webster; L. J. Palmer, Sonyea; L. R. Webber, Rochester; W. E. Stocking, Medina; D. P. Webster, Hilton; N. N. Leffler, Geneseo; Carl Webber, Rochester; W. G. Dodd, Canandaigua; A. George Tegg, Rochester.

The morning session included the routine business of the Association. Adjourned to meet at the veterinary hospital of Dr. L. R. Webber, on Andrew Street, at 1.30 P.M., at which place a very interesting clinic was held, resulting from the successful efforts of Dr. J. H. Taylor, Chairman of the Clinic Committee. A great number of operations were performed and witnessed by members of the Association.

W. E. STOCKING,
Secretary.

### NIAGARA AND ORLEANS COUNTIES VETERINARY MEDICAL ASSOCIATION.

THE annual meeting of the Niagara and Orleans Counties Veterinary Medical Association was held in the parlors of the Niagara House, Lockport, N. Y., August 26, 1902. The President, Dr. W. E. Stocking, called the meeting to order.

The roll-call found the meeting well attended, all parts of the counties being represented. The regular order of business was dispensed with, and a lively discussion followed on the subject of "Ringbone and Purpura Hemorrhagica," in which all participated.

The following officers were elected: President, M. D. Williams; Vice-President, James Martin; Secretary-Treasurer, Anderson Crowforth, Board of Censors: W. E. Stocking, G. C. Kesler, N. Hoffman, John O. Moore, John T. Liddle, H. S. Beebe, Wm. R. Hunter.

A. Crowforth,
Secretary.

